# How much does curation cost?

**DOI:** 10.1093/database/baw110

**Published:** 2016-08-08

**Authors:** Peter D. Karp

**Affiliations:** Bioinformatics Research Group, SRI International, 333 Ravenswood Avenue, Menlo Park, CA 94025, USA

## Abstract

NIH administrators have recently expressed concerns about the cost of curation for biological databases. However, they did not articulate the exact costs of curation. Here we calculate the cost of biocuration of articles for the EcoCyc database as $219 per article over a 5-year period. That cost is 6–15% of the cost of open-access publication fees for publishing biomedical articles, and we estimate that cost is 0.088% of the cost of the overall research project that generated the experimental results. Thus, curation costs are small in an absolute sense, and represent a miniscule fraction of the cost of the research.

## Perspective

In a recent article, Bourne *et al.* (1) argue that the recent flat research budgets for biomedical research imply that biomedical databases must chart a new course to explore new business models and methodologies. They are very concerned about the costs of databases in general, and of curation in particular. But to put these issues into a proper perspective, it is important to understand how much curation actually costs. Although many of us might expect that curation is quite expensive, we will show that its costs are quite modest on a per-article basis, and as a fraction of the cost of the original research.

We estimate the cost of curation for the EcoCyc database (2) using the following methodology. We want to be clear about this methodology to encourage other groups to perform similar estimates, and because this calculation involves a number of considerations. Our overall approach is to divide the cost of curation work over a given time period by the number of publications curated during that time period.

What do we mean by the cost of curation work? Our analysis considers curation only; we omit the costs of database and website operations, quality assurance, software development, outreach, preparation of publications, and bioinformatics research that are performed by EcoCyc and by other database projects. The reason we have excluded these other tasks is because they do not in fact involve curation per se. Furthermore, if we want to understand the benefits of replacing professional curation with say crowd-sourced curation or automated text mining, such replacement would not obviate other database costs such as outreach and website operations, so it is important to understand the costs of curation itself. We do include the costs of managing curators in our estimate of curation costs. Because in the EcoCyc project some of the preceding non-curation tasks are performed by curators, we have had to estimate what fraction of their time curators actually spend doing curation (that estimate ranges from 100% for some curators on the project to 80% for other curators). Note that costs will vary significantly across institutions because of variations in indirect costs (two EcoCyc curators work in Mexico and Australia, and NIH pays only an 8% indirect cost rate to foreign institutions) and in the cost of living (labor costs are quite low in Mexico).

What do we mean by number of publications? We have queried past versions of EcoCyc to determine the number of publications cited by the EcoCyc database in each version, using a program that interrogates every field in EcoCyc that could include a citation. These statistics are subject to possible over-counting and under-counting. Under counting could result if a curator neglected to cite within EcoCyc a curated publication, which we think would be extremely rare. Over-counting could occur if a curator cited an article that they thought relevant to say a gene that they were curating, when they had not actually curated the article. This situation does occur occasionally, but we think its incidence is <5%.

During the 5 years from May 2011 to 2016, the number of publications cited by EcoCyc increased by 9606 (from 21  448 to 31  054). Our curation costs during this period were $2.1M, yielding a curation-cost per publication of $219.

Thus, curation costs per publication were from 6 to 15% the cost of an open-access publication fee for publishing a biomedical article (open-access fees typically range from $1500 to $3500).

Furthermore, let us calculate the cost of curation as a fraction of the cost of performing the research. Let us postulate that an average NIH research grant (R01) has a budget of $250 000 per year, and produces one publication per year. The $219 curation cost is thus 0.088% of the cost of the overall research project—a minuscule price to pay for accurately curated and computable biological knowledge. We can also compare the curation cost of the research project to the cost of coffee breaks for the project. Imagine that a scientist who works 10 h per day on average takes one five-minute coffee break each day. They spend 0.83% of their time on such breaks. Thus, the curation cost is slightly more than one-tenth the cost of coffee breaks, a cost that is considered negligible.

We should not expect curation costs to be identical for every database because many factors will influence curation costs. Some databases may accept higher error rates than others [the error rate for EcoCyc curation has been estimated at 1.40% (3)]. Database curation procedures vary significantly, and we believe EcoCyc curation is likely to be relatively high on the scale of complexity because EcoCyc curators author long mini-reviews for genes and pathways, they extract a large number of database fields (350) for many different datatypes ranging from metabolic pathways to gene essentiality, and they capture molecular interactions at a high level of detail that enables generation of metabolic models from EcoCyc and capture of mechanisms of gene regulation for multiple types of regulation. On the other hand, EcoCyc curation costs are lowered by the preceding factors related to the non-U.S. groups who participate in EcoCyc. We estimate that if the non-US curation had been performed at a U.S. university, that the cost per publication would rise to approximately $320 per publication. This 50% increase, although significant, would not undermine the conclusions of this article: that curation costs are minute when compared to the cost of the research. In a future perspective we will examine whether the curation process is inefficient, and whether the other approaches suggested by Bourne *et al.*, such as direct curation by authors of a publication, are workable.

In summary, the $219 per publication cost of curation is a minuscule fraction (0.088%) of the cost of research—approximately one-tenth the cost of the coffee breaks for the researchers who performed the research. Open-access publication fees, which the scientific community apparently considers to be a reasonable tax on the research project, cost ∼1% of the budget of a research project—significantly more than the cost of curation.

## Funding

This work was supported by SRI International.

## Conflict of interest

None declared.
